# *De Novo* Assembly and Characterization of the Invasive Northern Pacific Seastar Transcriptome

**DOI:** 10.1371/journal.pone.0142003

**Published:** 2015-11-03

**Authors:** Mark F. Richardson, Craig D. H. Sherman

**Affiliations:** Deakin University, Geelong, Australia. School of Life and Environmental Sciences, Centre for Integrative Ecology, (Waurn Ponds Campus). 75 Pigdons Road. Locked Bag 20000, Geelong, VIC 3220, Australia; Wageningen UR Livestock Research, NETHERLANDS

## Abstract

Invasive species are a major threat to global biodiversity but can also serve as valuable model systems to examine important evolutionary processes. While the ecological aspects of invasions have been well documented, the genetic basis of adaptive change during the invasion process has been hampered by a lack of genomic resources for the majority of invasive species. Here we report the first larval transcriptomic resource for the Northern Pacific Seastar, *Asterias amurensis*, an invasive marine predator in Australia. Approximately 117.5 million 100 base-pair (bp) paired-end reads were sequenced from a single RNA-Seq library from a pooled set of full-sibling *A*. *amurensis* bipinnaria larvae. We evaluated the efficacy of a pre-assembly error correction pipeline on subsequent *de novo* assembly. Error correction resulted in small but important improvements to the final assembly in terms of mapping statistics and core eukaryotic genes representation. The error-corrected *de novo* assembly resulted in 115,654 contigs after redundancy clustering. 41,667 assembled contigs were homologous to sequences from NCBI’s non-redundant protein and UniProt databases. We assigned Gene Ontology, KEGG Orthology, Pfam protein domain terms and predicted protein-coding sequences to > 36,000 contigs. The final transcriptome dataset generated here provides functional information for 18,319 unique proteins, comprising at least 11,355 expressed genes. Furthermore, we identified 9,739 orthologs to *P*. *miniata* proteins, evaluated our annotation pipeline and generated a list of 150 candidate genes for responses to several environmental stressors that may be important for adaptation of *A*. *amurensis* in the invasive range. Our study has produced a large set of *A*. *amurensis* RNA contigs with functional annotations that can serve as a resource for future comparisons to other echinoderm transcriptomes and gene expression studies. Our data can be used to study the genetic basis of adaptive change and other important evolutionary processes during a successful invasion.

## Background

Invasive species occupy areas outside their historical range and often experience novel environmental conditions [1,2] that may result in strong selection on morphological and physiological traits [3]. Research has documented that adaptive change in response to novel environments is common during the invasion process [4–6]. Yet, the source of genetic or epigenetic variation underlying adaptive change during the invasion process remains largely uncharacterised [2,7], which has occurred in part, due to a lack of genomic information.

With the reduction in the cost of next generation sequencing technologies large quantities of genomic data can now be generated in a short time, which is particularly valuable for studies on non-model species [8]. Accordingly, we have seen several genomic resources created for invasive species over the past few years [9–13]. Transcriptome analyses in particular are useful for studying the molecular basis of responses to different environmental conditions. For example, thermal and salinity stress elicit diverged transcriptomic responses between two species of blue mussel (genus *Mytilus*), that may explain the invasive status of one and not the other [14,15]. Additionally, transcriptome resources have helped reveal substantial shifts in the expression of metabolism and cellular repair genes which may contribute to the increased dispersal ability of invasion front cane toads (*Rhinella marina*) [16]. Gene expression data can therefore provide valuable information to understand important evolutionary processes in invasion biology, especially because it links observable genetic changes to functional roles.

Marine ecosystems are particularly vulnerable to invasions, with coastal habitats among those harbouring the highest proportion of non-native species [17]. Arguably, one of the most successful invaders into Australian coastal waters over the past ~30 years is the northern Pacific seastar (*Asterias amurensis*). *A*. *amurensis*, is a benthic marine predator that has the potential to drastically alter native ecosystems and affect aquaculture industries [18,19]. In the national priority pests report, *A*. *amurensis* is ranked among the most potentially damaging invasive species in Australia [20]. After its introduction into southeast Tasmania in the early 1980s it spread northwards and established a large mainland population in Port Phillip Bay, Victoria, which was discovered in 1995. Recently, four further populations outside Port Phillip Bay have been discovered (Inverloch, San Remo, Tidal River, and Gippsland Lakes; all within the state of Victoria), suggesting that this species is currently undergoing a range expansion. Consequently, invasive *A*. *amurensis* populations provide an exciting opportunity to investigate the evolutionary response to novel environmental conditions and the underlying genetic basis of important processes in invasion ecology.

Here we report the sequencing of the *A*. *amurensis* bipinnaria larval transcriptome by RNA-Seq and the subsequent *de novo* assembly to produce a comprehensive set of reference contigs for gene discovery and gene expression studies. *A*. *amurensis* possess long-lived planktotrophic larvae that are capable of remaining in the water column for up to 112 days before settlement and development into juvenile seastars [21]. This early life history stage is highly dispersive and more susceptible to changes in environmental conditions than adults [22]. As such, the early larval stages are likely to be strongly influenced by novel selection pressures. The work presented here represents the first transcriptomic resource for this species. This resource will provide a valuable public dataset for future studies on the genetic basis of invasion and for comparisons to previously characterised echinoderm transcriptomes. Identification of a list of candidate genes that might respond to several environmental stressors, previously seen to be important in other marine invasions [14,15], can serve as a genetic resource to investigate ecological and evolutionary processes during the invasion of this species.

## Materials and Methods

### Sample collection and RNA isolation

*Asterias amurensis* adults were collected from Williamstown, Victoria, Australia in July 2012. Animal collection was conducted under the permission and in accordance with Victorian State Government Department of Primary Industries, Noxious Aquatic Species Permits NP152 and NP252. Adults were individually rinsed with UV-treated 1 μm filtered seawater in order to remove potential gamete cross-contamination and then induced to spawn by injecting with 1ml 10^−5^ M 1-methyladenine in filtered seawater into the coelom, as described in [23]. Males and females were spawned dry in separate containers; gametes rinsed and then re-suspended in 50 ml filtered seawater. The concentration of sperm for each male was determined from three replicate counts using an improved Neubauer haemocytometer and sperm standardized to 1 × 10^6^ sperm ml^-1^. Egg concentrations were assessed from three replicate counts using a Beckman multisizer™ 3 Coulter counter and standardized to 1 ×10^4^ eggs ml^-1^. Artificial fertilization was carried out in a total volume of 100 ml filtered seawater at 14°C, using 10,000 eggs from a single female and 100,000 sperm from a single male (sperm:egg ratio of 10:1). Gametes were left for 2 hours to fertilize at 14°C. Embryos were transferred into 1.5L containers (density of 5 larvae per ml) and cultured at 14°C for 10 days. Developing larvae were fed an algal diet of cultured *Chetocherous muleri* at 50,000 cells ml^-1^.

Cultured larvae were removed at the mid-bipinnaria larval stage (10 days post fertilization) and larval aliquots (approximately 2,000 individuals) were transferred to an 1.5 ml tube, gently spun to a pellet and the supernatant removed. The larvae pellet was immediately stored in Trizol Reagent (Invitrogen, USA), homogenized, flash frozen in liquid nitrogen and then transferred to a -80°C freezer for storage. Total RNA was extracted from this pooled sample of whole full-sib larvae using Trizol reagent according to the manufacturer’s instructions. Total RNA was further purified using an RNeasy spin column (Qiagen, USA) and the quality and quantity of total RNA measured using a NanoDrop 2000c spectrophotometer (Thermo Fisher Scientific Inc, USA).

### Sequencing, quality control and error correction

Sequencing and cDNA library preparation was conducted commercially at the Hawkesbury Institute for the Environment (University of Western Sydney, Australia). Briefly, 1ug of total RNA was used to construct a single polyA cDNA library using the Illumina TruSeq RNA protocol with the size selection step selecting for 200bp fragments. The amplified cDNA library was sequenced on one flow cell lane of the Illumina HiSeq-2000 platform, generating 100bp paired-end reads. Raw sequence reads were generated using the standard Illumina pipeline, exported in FASTQ format and deposited at the NCBI short read archive (SRA) under the Bioproject accession number [SRR1642063].

The raw sequence reads were filtered for quality in order to generate a high quality dataset for *de novo* assembly. Quality control steps were performed with the FASTX-Toolkit v0.0.13 (http://hannonlab.cshl.edu/fastx_toolkit/) and FastQC (http://www.bioinformatics.babraham.ac.uk/projects/fastqc/). First, raw reads containing adaptor contamination were discarded. Second, reads were filtered based on quality scores (Phred) and reads were discarded if 100% of bases in the read did not have a minimum Phred score of 20. Next, we computed the GC content distribution for all reads in the dataset. Random hexamer priming is known to introduce a GC content bias in the first 13 bases of Illumina RNA-Seq reads [24]. This bias might cause an imbalance in read coverage that persists through the assembly process, potentially affecting the quality of the assembly [25]. As our reads exhibited this uneven base content, we removed this bias by trimming the initial 15 bases from the reads.

For the successful implementation and generation of an accurate *de novo* assembly, the quality of the reads is paramount. While the quality control steps above can remove many assembly-confounding errors, certain specific sequence motifs can produce false positive base calling errors in Illumina HiSeq 2000 data [26,27]. To remove these systematic sequence read errors we utilized the Reptile v1.1 error correction pipeline (http://aluru-sun.ece.iastate.edu/doku.php?id=reptile) [28]. An initial optimization run was conducted to determine the configuration for error correction, as some Reptile parameters are dependent upon the data being used. A final run was conducted with the following parameters: *kmerLen* = 14, *T_expGoodCnt* = 8, *T_card* = 1, *MaxBadQPerKmer* = 6, *Qlb* = 67. Error corrected sequences were generated and used in the subsequent assembly. To assess the effect this step had on assembly quality we ran all subsequent assemblies and analyses on both the error-corrected and original (pre error correction) read sets.

### Digital normalization and *de novo* assembly

Our data exhibit very high sequence coverage, so in order to reduce computing power and the time needed for the *de novo* assembly we conducted digital normalization, which reduces the total number of reads to be assembled. Furthermore, assemblies generated with more than 60 million reads can lead to the accumulation of errors in highly expressed genes [29]. Digital normalization preferentially removes high abundance reads (reducing redundancy) while retaining read complexity and preserving low abundance reads [30]; it requires both the khmer (git://github.com/ged-lab/khmer.git) and screed software packages (git://github.com/ged-lab/screed.git). As recommended, we followed the single-pass digital normalization pipeline using normalize-by-median.py and–*C 20*, *-k 20* and–*x 4e9* parameters. These reduced read sets were assembled using Velvet v1.2.10 (https://github.com/dzerbino/velvet) [31] and Oases v0.2.8 (https://github.com/dzerbino/oases) [32]. We adopted an additive multiple *k*-mer approach [33], where *k*-mers ranged from 27 to 75 with a step of 4, so as to maximize contiguity in assembling highly expressed transcripts at high k-mers and sensitivity at low k-mers to assemble lowly expressed transcripts. Subsequently, these multiple *k*-mer assemblies were merged with another pass through Velvet and Oases at a *k*-mer of 27; only transcripts >100bp were kept. As anticipated, duplicate transcripts were present in the merged assemblies as a result of identical transcripts being produced at different *k*-mers. We used CD-HIT-EST v4.5.4 (http://weizhong-lab.ucsd.edu/cd-hit/) [34,35] to remove this redundancy (by matching sequences at the 95% level) and retain the longest possible transcripts (now termed contigs); at this stage we filtered out contigs <200bp.

To assess our assemblies we mapped the read set pre digital normalization back to the assembled contigs using BWA v0.7.7 (http://bio-bwa.sourceforge.net) [36]. We removed potentially spurious and uninformative contigs when each contig had an average read coverage of less than 5 ×, the majority of which were short, <500 bp. This generated a reduced set of contigs for both the error-corrected and original assemblies that were used in the following annotation pipeline. Contig statistics were computed with in house scripts. Finally, we used the python script KogBlaster.py v1.5 (https://bitbucket.org/beroe/mbari-public.git) [29] to search the assembled contigs against the 458 core eukaryotic genes (KOGs) from the CEGMA database (http://korflab.ucdavis.edu/datasets/cegma/) [37] and report completeness of the KOGs.

### Functional annotation

Functional annotation was carried out following a method described in [38]. The reduced set of contigs from the error corrected assembly was searched against the NCBI non-redundant protein database (NR) and UniProts’ Swiss-Prot and TrEMBl databases with BLASTX [39] using an E-value cutoff of 1.0 × 10^−3^; only the top 20 hits per query sequence were returned. We filtered matches to the NR database further to return only informative top hits by excluding hits to ‘predicted’ and ‘unknown’ proteins to enable more accurate mapping of Gene Ontology (GO) terms [40]. Top hits described as ‘predicted’ were kept for the species distribution to better represent the homology between sequences. GO terms were assigned to contigs, to infer functional annotations, based on the best hit from the databases with the following preference (Swiss-Prot, TrEMBL, and NR). We combined BLAST matches from the 3 databases, functional descriptions and associated GO terms into a master annotation metatable, using custom python scripts adapted from [38]. To avoid a representation of algal genes within the final transcriptome dataset (potentially arising through the assembly of genes expressed by sequenced gut contents) we removed any sequences that only had predominant hits to plant species or identified as constituents of: photosynthesis, Chloroplasts, Chlorophyll or the Calvin cycle during the annotation procedures.

The KEGG Automatic Annotation Server (KAAS) v1.6a (http://www.genome.jp/tools/kaas) [41] was used to annotate contigs with Kegg Orthology (KO) codes [42]. RepeatMasker v4.0.3 (http://www.repeatmasker.org) was used to search for repeating elements using the (22-4-2013) version of the RepBase database (http://www.girinst.org) [43]. RepeatMasker searches DNA sequences for interspersed repeats, including retroelements and DNA transposons and also reports simple repeats such as microsatellites. We ran RepeatMasker with default settings and the–*q*, quick search option with the species parameter set to echinoderms.

We identified candidate coding regions within assembled contigs by searching for ORFs containing the longest stretch of uninterrupted sequences between a start and stop codon, using TransDecoder r20131117 (http://transdecoder.sourceforge.net) with default options. This enables the further identification of informative functional contigs, even when they do not provide a significant match in the annotation process. Here, we consider a full-length contig to be those that show a complete CDS and at least partial 5’ and 3’ UTR sequences. The start and stop codons are used to define the boundary between the CDS and 5’ and 3’ UTRs. Contigs were considered to be partial CDSs if they contained, only a start or stop codon and a combination of 5’ or 3’ UTRs, or an uninterrupted chain of >100 amino acids and no start or stop codon. The CDS from the contigs were transcribed into proteins and searched against the Pfam databases (http://pfam.xfam.org) [44] to identify conserved protein domains using HMMER v 2.3.2 (http://hmmer.janelia.org) [45], with an E-value of 1.0 × 10^−5^. Contigs remaining without annotations or predicted CDS were further clustered with CD-HIT-EST at 90% similarity to compile a less redundant set of unannotated contigs which may represent novel *A*. *amurensis* sequences.

### Comparison to the Bat star, *Patiria miniata* proteins

The protein sequences of *P*. *miniata* were downloaded from (http:/spbase.org/) [46]. We performed reciprocal BLAST searches to identify putative orthologous genes following a method described [47]. Briefly, assembled *A*. *amurensis* contigs containing a CDS were compared to *P*. *miniata* protein using BLASTX. We then used tBLASTX to compare the *P*. *miniata* proteins to *A*. *amurensis* contigs used in the previous search. We retained only the best hit with an E-value cutoff > 1.0 × 10^−3^ and pairs of orthologous sequences were identified based on the reciprocal best matches. We randomly selected 200 reciprocal best hits, where both orthologs had annotation information and these were used to assess the efficacy of our annotation methods.

### Identification of candidate genes associated with environmental adaptation

We searched the annotated contigs for GO terms associated with: ‘response to heat’, ‘response to cold’, ‘response to stress’, ‘response to salt’, ‘osmotic stress’ and ‘oxygen binding’, to identify genes that might be associated with adaptation to novel marine environments in *A*. *amurensis*.

## Results and Discussion

### Sequencing and quality control

A cDNA library was constructed for the mid-bipinnaria larval stage of *A*. *amurensis*. We selected this developmental stage (10 days post fertilization) for two reasons: *i*) to avoid an overrepresentation of early developmental genes within the RNA sample and *ii*) to capture information on genes responding to environmental conditions experienced during the larval dispersive stage. Sequencing generated 58,776,662 pairs of 100 bp reads (11.7 Gbp). Quality control resulted in the removal of 26.9% of raw sequence reads leaving 35,078,206 pairs and 15,761,360 orphan reads, from which 1.28 × 10^9^ bases were removed during GC-content bias trimming. The second quality control phase corrected potential assembly-confounding systematic sequence read errors present in Illumina HiSeq-2000 data [26,27], which can improve the accuracy of assemblies [48]. This second phase identified and corrected 3,408,050 potential base call errors, accounting for 0.18% of bases in the digitally normalized read set. An error correction rate of 0.18% of bases is very small, so to examine the efficacy of error correction prior to *de novo* assembly and annotation we ran these procedures on both the error-corrected and original read sets.

### Transcriptome assembly

To reduce the time and computational power needed to assemble the transcriptome, we adopted a strategy that combines a digital normalization [30] step prior to assembly. The digital normalization strategy reduced both the error-corrected and original read data sets by 74.2%, resulting in 8,063,870 paired and 6,057,372 orphan reads that were used for assembly. An additive multiple *k*-mer approach with Velvet and Oases generated 713,013 pre-filtered transcripts (> 100 bp) with N50 of 1,907 bp for the error-corrected assembly and 725,467 pre-filtered transcripts (> 100 bp) with N50 of 2,000 bp for the original data set assembly. Digital normalization followed by assembly using Velvet and Oases has been shown to generate comparable results to assemblies with Trinity, while requiring substantially less computing resources [25].

**Fig 1:**
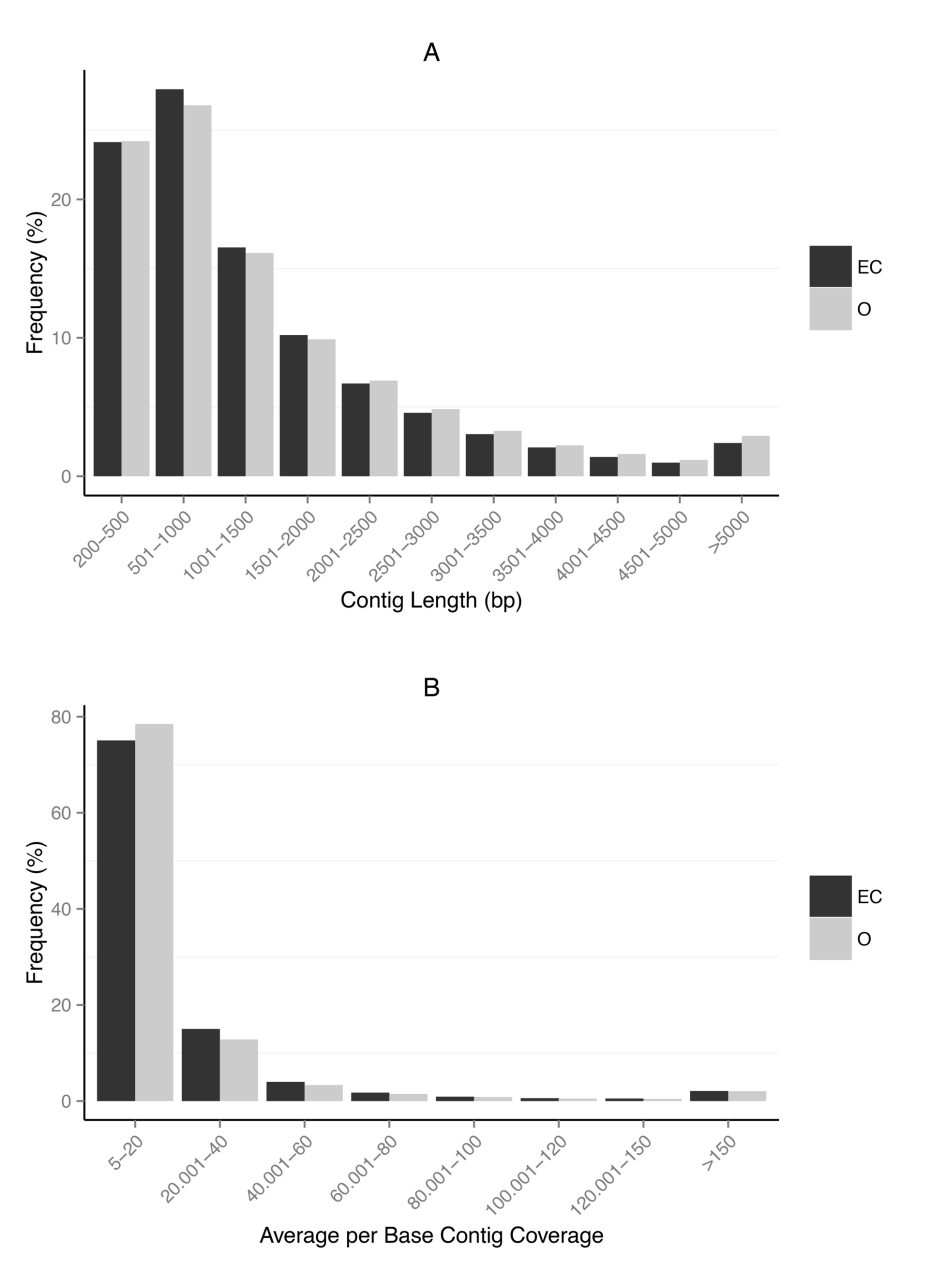
Length and coverage distributions of assembled contigs. (A) Contig length (bp) distribution for the error-corrected (EC) and original (O) datasets. (B) Contig coverage, calculated as average per base coverage across a contig, for the error-corrected (EC) and original (O) datasets.

Both assemblies exhibited redundancy so we used CD-HIT-EST to merge duplicate transcripts and retain the longest possible transcripts. Only transcripts >200bp and coverage >5× were kept. Filtering assemblies by length and coverage in this way has been demonstrated to effectively clean non-reference transcriptome assemblies [49]. In total, a redundancy-reduced assembly of 115,654 contigs with a N50 of 2,081 bp was generated for the error-corrected assembly and 123,388 redundancy-reduced contigs with a N50 of 2,229 bp for the original assembly (Table 1). Our assemblies show comparable summary statistics to other published non-model transcriptome assemblies in terms of N50, mean and median contig lengths [47,50,51]. Contigs from the error-corrected and original assemblies had total lengths of 1.6 × 10^6^ bp and 1.78 × 10^6^ bp, respectively. They exhibited similar characteristics in terms of mean and median lengths (error-corrected; mean = 1,383 bp, median = 954 bp; original; mean = 1,443 bp, median = 976 bp) and had a similar proportion of short (<300 bp) contigs. High levels of successful mapping to both assemblies’ contigs were observed (Table 1). However, mapping to the error-corrected contigs resulted in fewer discordant mappings (where one of a read pair maps to a contig but the other does not). This may be the result of the error-correction step producing fewer misassembles, spurious contigs and less partial gene fragments. Distributions of contig length and average base coverage for both assemblies are shown in Fig 1. Both assemblies again, exhibit similarity in the distribution of contig lengths, with the error-corrected assembly generating a larger proportion of contigs <2,000 bp, while the original assembly had fractionally more contigs > 2,000 bp. Yet, the error-corrected assembly produced contigs with higher average per-base coverage.

**Table 1:** Assembly summary statistics for the two different invasive *A*. *amurensis* bipinnaria larval *de novo* assemblies. (bp) refers to length in base pairs.

	Error corrected assembly	Original assembly
Number of contigs	115,654	123,388
Total contig length (bp)	1.60 × 10^6^	1.78 × 10^6^
Mean contig length (bp)	1,383	1,443
Median contig length (bp)	954	976
Minimum contig length (bp)	200	200
Maximum contig length (bp)	26,819	27,497
N50 (bp)	2,081	2,229
Contigs <300 bp (%)	9.68	9.66
Alignment rate (%)	94.05	93.82
Discordant mappings (%)	4.90	4.99

We evaluated the representation of conserved core eukaryotic genes (KOGs) to assess the completeness of the two assemblies. We found that 457 (99.8%) of the KOGs had at least one hit in both of the error-corrected and original assemblies. A total of 397 (86.7%) and 394 (86.0%) KOGs, for the error-corrected and original respectively, had successful alignments (Table 2). Of the successful alignments, fewer full-length alignments were reported for the original (92.9%) than error-corrected (94.2%) assembly. Additionally, fewer potential nonsense alignments were reported for the error-corrected assembly (0.06%), as compared to 0.08% in the original. This shows the transcriptome assembly strategies we adopted were able to successfully assemble a majority of contigs that represent conserved KOGs. This is similar to representation of KOGs in other recent transcriptome assemblies [11,52]. While the error-corrected read set produced a better quality assembly in terms of KOG representation (0.7% improvement in KOGs identified; 1.5% improvement in full-length KOG assemblies and 20% fewer discordant mappings) the magnitude of difference between the assembly strategies was small. This suggests adopting an error-correction strategy before *de novo* assembly may not always generate substantially better *de novo* assemblies and should be assessed on a species by species basis. However, with transcriptome assembly of non-model species, the goal is generally to build the most comprehensive set of genes for use in further experimental work. Consequently, even marginal improvements in generating more full-length gene assemblies may be beneficial. As such, contigs from the error-corrected assembly were used for all subsequent analysis.

**Table 2:** Summary of alignments to the 458 core eukaryotic genes (KOGs). Percentage (%) of total KOGs in parentheses.

	Error corrected assembly	Original assembly
KOGss with hits	457 (99.8)	457 (99.8)
Successfully aligned	397 (86.7)	394 (86.0)
Full-length alignments	374 (81.7)	366 (79.9)
Potential nonsense alignments	28 (0.06)	35 (0.08)

### Functional annotation

Homology-based functional annotation was carried out on the error-corrected set of 115,654 contigs utilizing BLASTX searches against the NCBI non-redundant protein (NR), Swiss-Prot and TrEMBl databases. A total of 41,667 contigs had a match to a known protein within the three databases, covering 36% of all contigs (Table 3). Of these, we were able to map Gene Ontology (GO) terms to 87.8% of matches, comprising 31.6% of all contigs. The largest annotated contig was 26,819 bp, which corresponds to the axonemal dynein heavy chain, a motor protein that causes sliding of microtubules in cilia and flagella. The 73,987 (64%) contigs that did not produce a BLASTX match to a known protein are predominantly shorter (mean 964.9 bp; median 720 bp) than those annotated (mean 2,042 bp and median 1,572 bp, respectively). The group of unannotated contigs still likely contains some biologically relevant contigs that code for novel proteins and polyadenylated non-coding RNAs without similar sequences within the databases. However, our annotation rate is within the range reported (20–40%) for several other *de novo* transcriptome assemblies in non-model species [47,53–55]. While we successfully annotated > 41, 000 contigs, this will be an over-representation of the true number of expressed *A*. *amurensis* genes. This likely occurs due to annotating contigs separately that: *i*) belong to the same multi-domain containing genes, *ii*) are fragments of the same gene and *iii*) constitute separately assembled allelic variants and isoforms of the same gene. We estimate 11,355 genes are expressed in *A*. *amurensis* bipinnaria larvae (based on the number of unique annotated genes), which is comparable to gene expression levels reported for several developmental stages (~11,500) in the purple sea urchin, *Strongylocentrotus purpuratus* [56].

**Table 3:** Summary of BLASTX annotations.

	Number of contigs	Percentage (%)
With BLASTX match	41,667	36
- with GO annotation	36,467	31.6
- without GO annotation	5,200	4.5
Without BLASTX match	73,987	64

**Fig 2:**
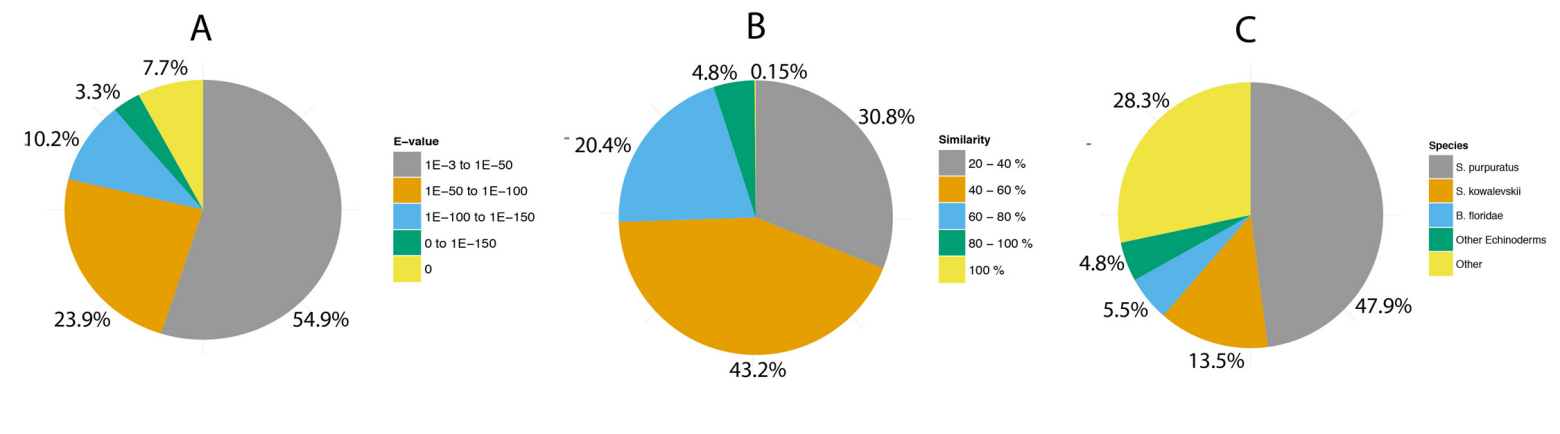
BLASTX annotation results. (A) Distribution of E-values for BLASTx top hits for each contig with a cutoff E-value of < 1.0 × 10^−3^. (B) Similarity distribution based on the percent (%) match of the BLASTX top hits and each query contig. (C) Filtered species distribution of BLASTX top hits where hits have a > 60% similarity to query sequences containing >100 amino acid residues. ‘Other’ represents the grouping of species with low numbers of hits to query contigs together.

The frequency distribution of top hit E-values shows that 45.1% of annotated contigs exhibit strong homology with a matched database protein (E-value < 1.0 × 10^−50^), while the majority of sequence matches (54.9%) had an E-value range of 1.0 00D7 10^−50^–1.0 × 10^−3^ (Fig 2A). Of the annotated contigs 25.4% (10,569) had a percentage similarity > 60% to a matched database protein, while 69.1% (28,789) had a similarity of > 40% and 30.8% (12,827) had a similarity between 20–40% (Fig 2B). Although our contigs exhibit a high proportion of strong matches (E-value < 1.0 × 10^−50^; 45.1%), a smaller percentage of matches (25.4%) cover the majority of the contig sequence. This likely arises through BLAST matches to sequences sharing short, highly conserved functional domains having statistically stronger matches to our sequences. Consequently, the similarity between some matches to sequences of different species may not represent true orthology. A filtered species list is proposed to be better able to reconcile interspecific contig homology as only longer alignments with a high sequence similarity are retained [57]. As such, we filtered the species-hit distribution to remove lower similarity BLASTX matches, retaining only those with similarity of > 60% and where a contig contains > 100 amino acid residues (n = 8,544)(Fig 2C). This filtered species hit distribution shows the most represented species, with 47.9% of top hits, being the purple sea urchin *S*. *purpuratus*, which has the most extensive genomic information for echinoderms. The next most represented species is an acorn worm, *Saccoglossus kowalevskii* (13.5%), which belongs to the Hemichordata phylum and is closely related to the Echinodermata. A further 4.8% of top hits come from other species belonging to the Echinodermata, with the most represented of these being the sea stars, *Patiria pectinifera* and *P*. *miniata*. Only a small number of top hits are to previously described *A*. *amurensis* proteins (28 in total), although this is expected due to the limited genomic resources for this species. This filtered species list of top hits reveals strong homology between our *A*. *amurensis* assembled contigs and Echinodermata proteins. While 52.7% of annotated sequences match echinoderm proteins the proportion to closely related Asteroids is small (< 4.8%) and is most likely due to insufficient sequences from phylogenetically closely related species in the searched databases [47]. Furthermore, the BLASTX annotation procedure is biased by the completeness of genome annotations for each respective genome within the searched databases [58]. Thus the majority of our BLAST hits are to *S*. *purpuratus* sequences and not closely related Asteroidia. These issues are an inherent problem with this method of annotating sequences to the available protein databases, although this approach is still used extensively [16,25,59,60] and often represents the best available annotation method for non-model species where there are little or no genomic resources for closely related species. However, the Echinobase database contains sequence data for several echinoderm species, including another seastar and we used this data to identify Asteroid orthologs and examine our gene annotations (see below).

### Comparative annotation validation to the Bat star, *Patiria miniata* proteins

The Bat star, *Patiria miniata* represents the closest echinoderm species for which extensive genomic and transcriptomic data is available [46], allowing direct comparison to the *A*. *amurensis* sequences produced here. Reciprocal BLAST searches revealed 99.2% (41,365) of assembled *A*. *amurensis* proteins had significant matches to 47% (14,032) of *P*. *miniata* proteins and 92.9% (27,692) of *P*. *miniata* proteins had significant matches to 30.1% (12,535) of *A*. *amurensis* proteins. In total, 9,739 best matches were common to both BLAST searches, representing putative orthologs between the two species (S1 Table). This is potentially an underestimate of the actual number of orthologs, as our data set contains both full and partial protein sequences that map to several *P*. *miniata* proteins. We annotated 2,423 *A*. *amurensis* contigs whose corresponding *P*. *miniata* ortholog did not have annotation information, while only 125 *P*. *miniata* proteins had annotation information when the *A*. *amurensis* ortholog did not. To determine the accuracy of our annotation pipeline we manually compared the annotations of 200 randomly selected putative orthologs that both had annotation information. 89.5% (179) of our *A*. *amurensis* annotations were positives (i.e. matched correctly with those of the *P*. *miniata* orthologs), while 10.5% (21) exhibited discrepancies. The high number of positive annotations reveals the efficacy of our annotation methods and validity of our dataset for future studies. The discrepancy in annotations may represent mis-annotation due to BLAST matches against short protein domains or be due to differences in gene nomenclature. For example, we annotate an *A*. *amurensis* protein as Serine incorporator 5, while the *P*. *miniata* ortholg is identified as a Serine incorporator 3. Such mis-annotations are not unusual from electronic annotation pipelines and can only be resolved through further manual curation.

### Gene Ontology (GO) and KEGG annotation

**Fig 3:**
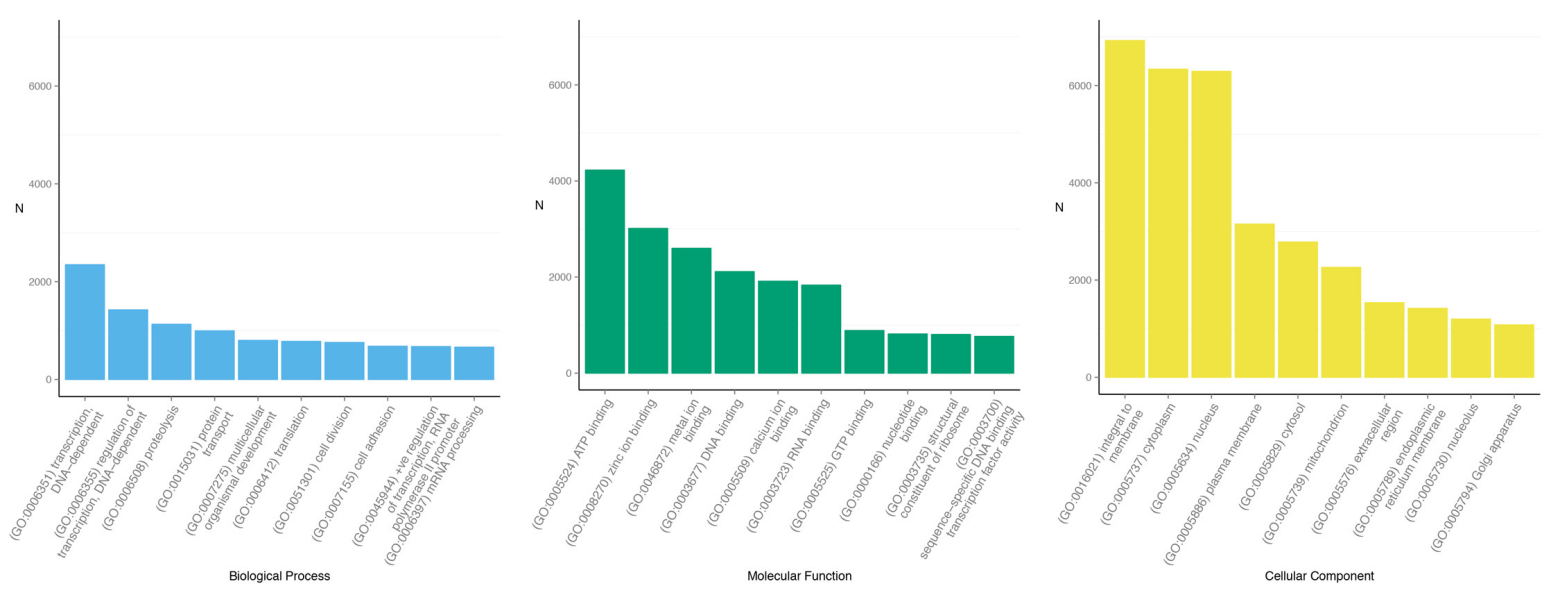
Gene Ontology (GO) annotations. The top 10 represented GO terms for each of the GO categories: Biological Process, Molecular Function and Cellular Component. GO functional annotations are derived from similarity to the protein databases (Swiss-Prot, TrEMBL and NCBI’s non-redundant database).

**Fig 4:**
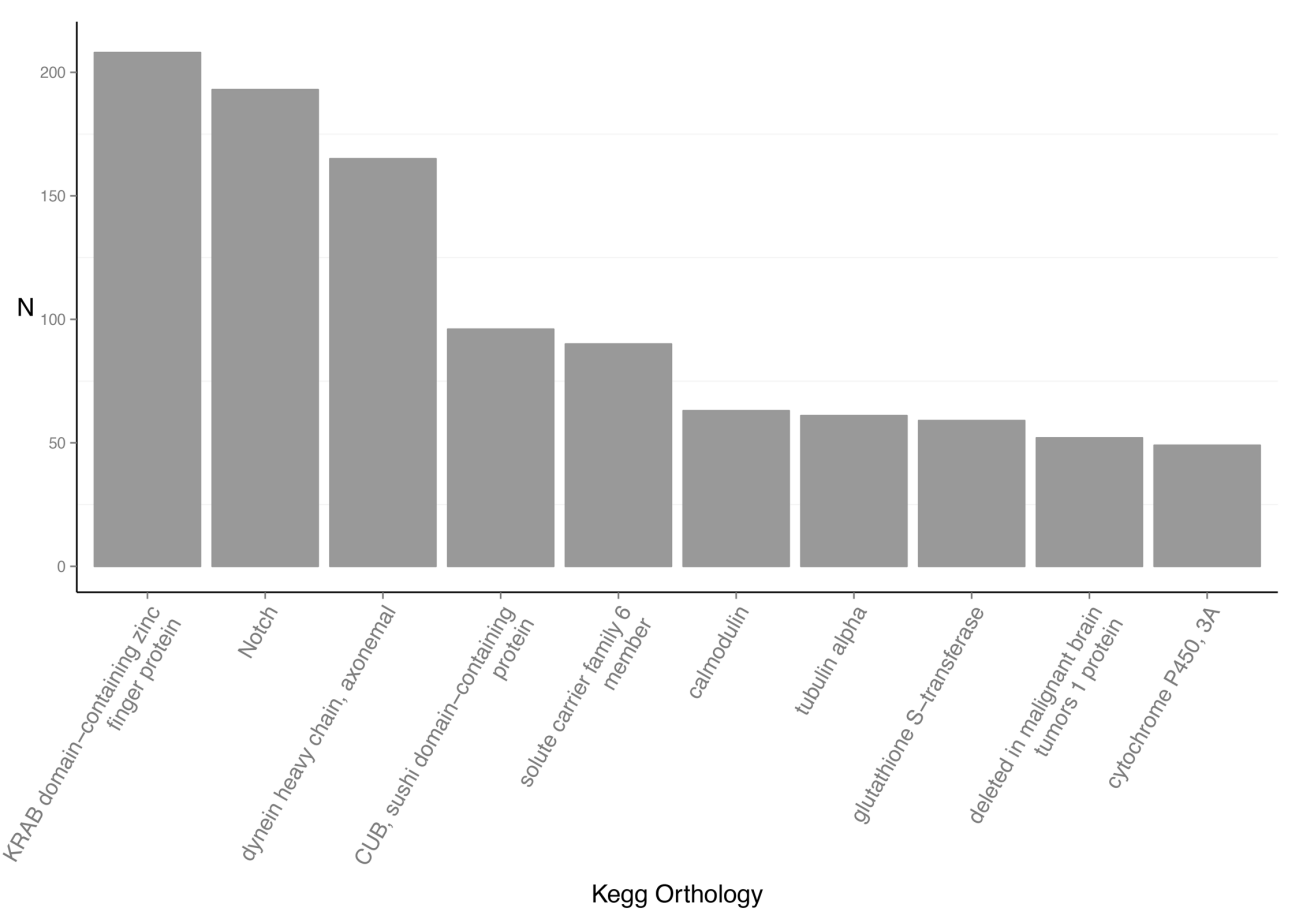
Kegg Orthology (KO) annotations. The top 10 represented KO terms from the KEGG Automatic Annotation Server (KAAS) annotation results.

To functionally categorize the *A*. *amurensis* contigs, we mapped the associated GO terms to the 41,667 contigs that had BLAST matches. In total, 258,322 GO terms were mapped to 36,465 annotated contigs. GO terms are divided into three GO categories, biological process, molecular function and cellular component, each containing 7,144; 2,704 and 1,091 unique GO terms, respectively. The top 10 GO assignments for each of the three categories are detailed in Fig 3. The top represented GO terms for biological process were transcription (2,346), regulation of transcription (1,423) and proteolysis (1,128). For molecular function the top represented terms are from binding domains; ATP binding (4,226), zinc ion binding (3,012) and metal ion binding (2,598). Lastly, the top cellular component GO terms were, integral to membrane (6,927), cytoplasm (6,338) and nucleus (6,294). We used the KEGG Automatic Annotation Server (KASS) to provide KEGG Orthology (KO) annotations to the annotated contigs. This resulted in 5,533 unique KO annotations to 24,929 contigs. The top 10 represented KO annotations are provided in (Fig 4) with the most represented being the KRAB-domain containing zinc finger protein (208), Notch (193) and DNAH: dynein heavy chain, axonemal (165).

### Identification of coding sequences and protein domains

Following the homology-based BLAST annotation process, 73,987 contigs (64% of all contigs) did not have a significant match to a protein in any of the three databases. However, it is likely that some of these contigs are derived from protein-coding genes, representing novel *A*. *amurensis* mRNAs. These contigs may have failed to get a significant BLAST hit due to a truncated coding sequence (CDS) or their relatively short overall length compared to the annotated contigs, potentially arising from incomplete assembly. To identify unannotated potential protein-coding genes we predicted open reading frames (ORFs) and extracted amino acid sequences for the unannotated contigs. This revealed 3,176 contigs (4.29%) that contained putative ORFs of > 100 amino acids in length. To further evaluate the quality of our annotated contigs we performed the same ORF searching on the previously annotated contigs. Of the annotated contigs, 36,175 (86.7%) contained a putative CDS larger than 100 amino acids, giving a combined total of 39,351 predicted proteins in the error-corrected assembled contigs. This set of predicted proteins contains 18,319 unique proteins with homology-based annotations. This is larger than our estimate for unique genes expressed (11,355) with the redundancy attributable to isoform and allele specific assembly during *de novo* assembly and potential separate assembly and annotation of multi-domain proteins.

To provide further functional information, the translated ORFs were searched against the Pfam database to identify conserved protein domains. In total, 91,083 protein domains were identified, representing 4,762 unique domains. The top represented domains (Table 4) were the Zinc finger, C2H2 type, Ankyrin repeat domain and Epidermal growth factor-like (EGF) domains. The zinc finger C2H2 domain is an ubiquitous interacting domain, reported to be involved in sequence-specific DNA binding, RNA binding, as well as mediating protein interactions [61]. This method of identifying functional roles is also prone to the problems associated with BLAST searches, i.e. preferentially identifying short sequence matches, and electronic annotation discussed previously, see [62]. As such, it should only be considered a preliminary analysis of putative function.

**Table 4:** The top 10 represented Pfam domains from the protein domain annotations.

Pfam domain	Number of contigs
zfC2H2: Zinc finger, C2H2 type	5,363
Ank: Ankyrin repeat domains	4,886
EGF: Epidermal growth factor-like domains	3,447
LRR: Leucine-rich repeat motifs	3,029
TPR: Tetratricopeptide-like repeats	2,847
EFhand: helix-loop-helix structural domains	2,085
WD40: WD40 repeat containing domain	1,980
RRM: RNA recognition motif	1,771
Pkinase: Protein kinases	1,710
Ig: Immunoglobulin domain	1,291

### Transposable elements

To further explore the unannotated contigs (73,987), we assessed the representation of repeating elements including retroelements and DNA transposons in the assembled *A*. *amurensis* transcriptome. Such transposable elements (TE) are proposed to have important roles in the adaptive process of invasive species in response to different environments, either through the maintenance of genetic variation or contribution to phenotypic plasticity [63,64]. Additionally, mounting evidence indicates TEs are under selection following environmental stress (both abiotic and biotic) and that TE activity may have facilitated adaptation across many taxa [65–68].

In our data, retroelements constitute the majority (annotated, 78%; unannotated, 72%) of TEs compared to DNA transposons (annotated, 20%; unannotated, 27%). Both sets of contigs exhibit similar representation of retroelement classes (S2 Table), however, retroelements are proportionately less abundant in the unannotated than annotated set of contigs, despite fewer retroelements overall reported for the annotated set (S2 Table). TEs are much less abundant in *A*. *amurensis* (~0.34%) than the sea urchin *Evechinus chloroticus* transcriptome (~2–3%) [52]. The representation of TE classes is similar between and *A*. *amurensis* and *E*. *chloroticus* although there are differences in DNA transposon diversity, particularly PiggyBac and Tourist/Harbinger, which show opposite abundances. The estimates of the number and diversity of TEs present within the *A*. *amurensis* transcriptome presented here can serve as a useful start for further studies investigating a potential role of TEs during the *A*. *amurensis* invasion and for comparisons to other invasive species TE diversity estimates. TEs identified from RNA-Seq data may be particularly important as they are likely to include TEs close to genes and regulatory regions, which are more likely to be involved in rapid adaptation [64].

### Identification of candidate genes for environmental adaptation

To identify genes that may be involved in environmental adaptation in the invasive range we searched the annotated contigs’ GO terms for: ‘response to heat’, ‘response to cold’, ‘response to stress’, ‘response to salt’, ‘osmotic stress’ and ‘oxygen binding’. Previous research has shown that environmental perturbations have elicited gene expression responses in genes linked to these GO categories [14,15,69,70]. In total, we identified 150 genes (S3 Table) that will serve as *a priori* candidates to investigate how populations of *A*. *amurensis* have adapted to novel environmental conditions across their native and invasive range.

## Conclusions

Using high throughput paired-end sequencing of RNA extracted from mid-bipinnaria larvae followed by *de novo* assembly we derived a dataset comprising 115,654 contigs from the *A*. *amurensis* transcriptome. Of these, we functionally annotated 41,667 through significant matches to three protein databases. These annotated contigs were assigned Gene Ontology and Kegg Orthology terms and annotated with Pfam protein domains to provide additional information. Overall, we identify and provide functional information for 18,319 unique proteins, comprising at least 11, 355 expressed genes, with the remainder likely constituting gene isoforms and allelic variants. Of the annotated genes at least 9,739 are orthologs to *P*. *miniata* proteins. This data allowed the construction of a list of candidate genes that might respond to changing environmental conditions experienced during the dispersive phase in this species and will form the basis for further investigation. The relatively recent invasive history and contemporary range expansion of *A*. *amurensis* provides exciting opportunities to study the genetic basis of evolutionary adaptation during the invasion process. The construction of this larval transcriptome can serve as a genetic resource to investigate interesting questions in regards to ecological and evolutionary processes, such as the genetic and plastic basis of rapid adaptation and evolution occurring during the invasive range expansion.

## Supporting Information

S1 Table Gene annotations for identified orthologs between *A*. *amurensis* and *P*. *minata*. Annotations from this study and for *P*. *miniata* proteins from echinobase are shown.
(XLSX)

S2 Table Summary of repeating elements. Repeating elements identified with Repeatmasker in the annotated and unannotated contig sets.
(DOCX)

S3 Table Candidate genes with a putative response to environmental change. Gene name and associated Gene Ontology terms are listed.
(XLSX)
