# Supplementary material for: De Novo Assembly and Characterization of the Invasive Northern Pacific Seastar Transcriptome
Source: PLoS One. 2015 Nov 3;10(11):e0142003. doi: 10.1371/journal.pone.0142003 (PMC4631335; doi:10.1371/journal.pone.0142003)
Supplement: S2 Table — Repeating elements identified with Repeatmasker in the annotated and unannotated contig sets. (DOCX) [file pone.0142003.s002.docx]

**S2 Table. Summary of repeating elements**

|  | **Number of elements** | **Percentage of total sequence (%)** |
| --- | --- | --- |
| **Retroelements** | 744 (998) | 0.15 (0.11) |
| SINEs: | 430 (857) | 0.04 (0.10) |
| -Penelope | 0 (0) | 0 (0) |
| LINEs: | 205 (96) | 0.05 (0.01) |
| -CRE/SLACS | 0 (0) | 0 (0) |
| -L2/CR1/Rex | 146 (80) | 0.04 (0.01) |
| -R1/LOA/Jockey | 9 (3) | ~0 (~0) |
| -R2/R4/NeSL | 1 (0) | ~0 (0) |
| -RTE/Bov-B | 49 (13) | 0.01 (~0) |
| -L1/CIN4 | 0 (0) | 0 (0) |
| LTR elements: | 109 (45) | 0.06 (~0) |
| -BEL/Pao | 13 (1) | 0.1 (~0) |
| -Ty1/Copia | 0 (0) | 0 (0) |
| -Gypsy/DIRS1 | 95 (38) | 0.05 (~0) |
| -Retroviral | 0 (0) | 0 (0) |
| **DNA transposons** | 186 (377) | 0.03 (0.05) |
| -hobo-Activator | 27 (65) | ~0 (0.01) |
| -Tc1-IS630-Pogo | 14 (56) | ~0 (0.01) |
| -En-Spm | 0 (0) | 0 (0) |
| -MuDR-IS905 | 0 (0) | 0 (0) |
| -PiggyBac | 0 (1) | 0 (~0) |
| -Tourist/Harbinger | 80 (192) | 0.01 (0.02) |
| -Other (Mirage. P-element, Transib) | 0 (0) | 0 (0) |
| Unclassified | 21 (10) | ~0 (~0) |
| **Total interspersed repeats** | **951 (1,385)** | **0.18 (0.16)** |
|  |  |  |
| Small RNA | 44 (13) | 0.03 (~0) |
| Satellites | 0 (0) | 0 (0) |
| Simple repeats | 21,714 (40,512) | 1.39 (3.25) |
| Low complexity | 3,237 (4,777) | 0.54 (1.03) |
|  |  |  |
| **Total** | 25,946 (46,687) | 2.13 (4.44) |

~ Denotes approximate value. Values in parentheses correspond to those from the set of assembled contigs for which there were no matches within any of the protein databases. Values not in parentheses are derived from the set of contigs with matches in the protein databases.
